# Antioxidative Efficacy of a *Pistacia Lentiscus* Supplement and Its Effect on the Plasma Amino Acid Profile in Inflammatory Bowel Disease: A Randomised, Double-Blind, Placebo-Controlled Trial

**DOI:** 10.3390/nu10111779

**Published:** 2018-11-16

**Authors:** Efstathia Papada, Alastair Forbes, Charalampia Amerikanou, Ljilja Torović, Nick Kalogeropoulos, Chara Tzavara, John K. Triantafillidis, Andriana C. Kaliora

**Affiliations:** 1 Department of Dietetics and Nutritional Science, School of Health Science and Education, Harokopio University, 17671 Athens, Greece; efpapada@gmail.com (E.P.); amerikanou@windowslive.com (C.A.); nickal@hua.gr (N.K.); htzavara@med.uoa.gr (C.T.); 2 Norwich Medical School, University of East Anglia, Bob Champion Building, James Watson Road, Norwich NR4 7UQ, UK; Alastair.Forbes@uea.ac.uk; 3 Department of Pharmacy, Faculty of Medicine, University of Novi Sad, 21000 Novi Sad, Serbia; ljilja.torovic@izjzv.org.rs; 4 Inflammatory Bowel Disease Unit, IASO General Hospital, 15562 Athens, Greece; jktrian@gmail.com

**Keywords:** oxidative stress, amino acids, inflammatory bowel disease, Crohn’s disease, ulcerative colitis, *pistacia lentiscus* (mastiha)

## Abstract

Oxidative stress is present in patients with Inflammatory Bowel Disease (IBD), and natural supplements with antioxidant properties have been investigated as a non-pharmacological approach. The objective of the present study was to assess the effects of a natural *Pistacia lentiscus* (PL) supplement on oxidative stress biomarkers and to characterise the plasma-free amino acid (AA) profiles of patients with active IBD (Crohn’s disease (CD) *N* = 40, ulcerative colitis (UC) *N* = 20). The activity was determined according to 5 ≤ Harvey Bradshaw Index ≤ 16 or 2 ≤ Partial Mayo Score ≤ 6. This is a randomised, double-blind, placebo-controlled clinical trial. IBD patients (*N* = 60) were randomly allocated to PL (2.8 g/day) or to placebo for 3 months being under no treatment (*N* = 21) or under stable medical treatment (mesalamine *N* = 24, azathioprine *N* = 14, and corticosteroids *N* = 23) that was either single medication (*N* = 22) or combined medication (*N* = 17). Plasma oxidised, low-density lipoprotein (oxLDL), total serum oxidisability, and serum uric acid were evaluated at baseline and follow-up. OxLDL/LDL and oxLDL/High-Density Lipoprotein (HDL) ratios were calculated. The plasma-free AA profile was determined by applying a gas chromatography/mass spectrometry analysis. oxLDL (*p* = 0.031), oxLDL/HDL (*p* = 0.020), and oxLDL/LDL (*p* = 0.005) decreased significantly in the intervention group. The mean change differed significantly in CD between groups for oxLDL/LDL (*p* = 0.01), and, in the total sample, both oxLDL/LDL (*p* = 0.015) and oxLDL/HDL (*p* = 0.044) differed significantly. Several changes were reported in AA levels. PL ameliorated a decrease in plasma-free AAs seen in patients with UC taking placebo. In conclusion, this intervention resulted in favourable changes in oxidative stress biomarkers in active IBD.

## 1. Introduction

Inflammatory bowel disease (IBD) is a chronic gastrointestinal disease that includes Crohn’s disease (CD) and ulcerative colitis (UC). Patients face several intestinal and extraintestinal complications (e.g., hepatobiliary and ocular complications). In addition, IBD patients in relapse are at increased risk of acute arterial events even at a young age [1] and at increased risk of acute myocardial infarction and heart failure even if they do not meet the traditional risk factors for cardiovascular disease [2].

Oxidative stress has been proposed as both a putative causal and perpetuating factor for IBD [3]. Oxidative stress plays a critical role in the pathogenesis, progression, and severity of IBD, serving as an immunoregulatory factor. Chronic inflammation of the intestinal mucosa stimulates excessive reactive oxygen species (ROS)/reactive nitrogen species (RNS) production leading to oxidative stress [4,5]. Alterations in RNS levels recruit phagocytes and Nitric Oxide Synthases (NOS), which affect the nitric oxide levels of the intestinal mucosa [6], lead to overproduction of cytotoxic reactive oxygen metabolites, and inhibit the antioxidative system. The above results in oxidative injury of the intestinal mucous membrane [7]. Additionally, the link between IBD severity and duration with the pathogenesis of atherosclerosis [8] has been explained by the release of inflammatory cytokines that lead to endothelial dysfunction [9].

Amino acids (AAs) control and protect intestinal health, and not only act as substrates for proteinosynthesis in intestinal mucosal cells [10], but also as regulators of major metabolic pathways [11,12]. Their protective function may be linked to intestinal epithelial cells apoptosis and proliferation, as well as inhibition of inflammation and oxidative stress through blocking of the NF-κB signaling pathway [13]. Recently, it has been shown that variations in the levels of some AAs may be involved in the pathogenesis of IBD [14]. Data on circulating AAs in active IBD patients are limited and sometimes conflicting; however, it seems that the AA profile differentiates IBD patients from healthy controls, pointing towards a link between the AAs and the disease process [15,16,17]. Aminograms and multivariate indexes using AA molar ratios have been proven to be useful in reflecting the nutritional status, assessing disease activity, and monitoring disease progression [15,18].

*Pistacia lentiscus* L. var latifolius Coss or *Pistacia lentiscus* var. Chia is an evergreen shrub of the *pistacia* genus that is cultivated for its aromatic resin mainly on the Greek island of Chios. Previous studies of *Pistacia lentiscus* resin have documented the presence of phenolics [19], terpenes [20,21], their bioavailability in humans [22], as well as antioxidant [23], anti-inflammatory [24], lipid-lowering [25], and immunomodulating properties [26,27] of this natural product.

The European Medicines Agency has recognised *Pistacia lentiscus* resin as a herbal medicinal product with the following indications: (a) mild dyspeptic disorders, (b) symptomatic treatment of minor inflammations of the skin, and (c) as an aid in the healing of minor wounds [28].

In previous research, it has been reported that a 3-month intervention with a supplement containing the natural resinous product of *Pistacia lentiscus* (the supplement herein given as PL), adjunctive to stable medical treatment, regulated faecal biomarkers and improved quality of life in active IBD patients compared with a placebo [29]. Herein, the aim was to explore the effects of this intervention on biomarkers of oxidative stress in active IBD. Additionally, another study in healthy adults has shown that AA profiles are modified in response to acute supplementation with PL resin [30]. AAs are key regulators in metabolic pathways that affect intestinal health, and are also implicated in oxidative stress and inflammatory cytokines production [31,32]. Therefore, a second aim was to assess whether PL administration could have an impact on the health of patients reflected in the plasma-free AA profile after an assessment of AAs in the diet.

## 2. Materials and Methods

### 2.1. Study Design

Τhe Harokopio University Ethics Committee (49/29-10-2015) reviewed and approved the protocol, and the trial was conducted according to the rules of the Declaration of Helsinki of 1975 as revised in 2008. All subjects gave their informed consent before they participated in the study. The trial was registered with ClinicalTrials.gov, where the full trial protocol can be accessed (Identifier: NCT02796339). Patients were recruited between May 2016 and June 2017. Follow-up visits were completed on September 2017. The study took place in Athens, Greece.

**Figure A1:**
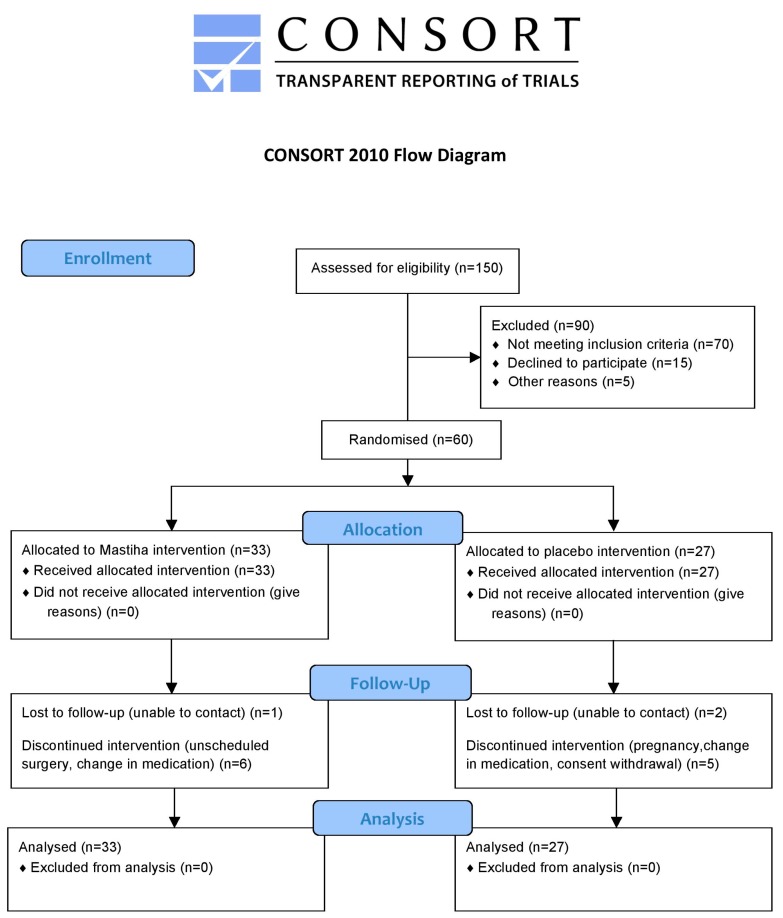
CONSORT 2010 flow diagram.

IBD patients in relapse were enrolled in this randomised, double-blind, placebo-controlled, parallel-group clinical trial (Appendix A Figure A1). The primary endpoint was attenuation of oxidative stress biomarkers. The secondary endpoints were plasma-free AAs profile and quality of life assessment. In brief, IBD patients in relapse, with endoscopically confirmed UC or CD, were enrolled based on certain inclusion and exclusion criteria. In detail, both males and females, of age ranging between 18 and 67 years old, with a diagnosis of IBD established by endoscopy, and with consistent histology and clinical course were included. To avoid hospitalisation, surgeries, and major changes in drug treatments, the study was designed to include moderately active patients. Active disease was assessed by applying the Harvey Bradshaw Index (5 ≤ HBI ≤ 16) [33] for CD patients or the Partial Mayo Score (2 ≤ PMS ≤ 6) for UC patients [34]. Medication included treatment with steroids for at least 2 weeks before the initiation of the trial, mesalamine and mesalamine analogues for 4 weeks, and immunosuppressants for 8 weeks before the start of the trial. Patients were excluded from trial enrollment in the case of positive stool culture for enteric pathogens or Clostridium difficile toxin or when treated with antibiotics during and 2 months prior to screening. Also, patients who had undergone bowel surgery ≤3 months prior to screening or a planned elective surgery or hospitalisation during the study were excluded. Additionally, clinically significant short bowel syndrome, the presence of an intra-abdominal abscess or a fistula with clinical or radiological evidence of an associated abscess, ileostomy, and colostomy were exclusion criteria. Enteral or parenteral nutrition, alcohol or drug abuse, vitamin or inorganic supplement use <6 months prior to screening, a vegan or macrobiotic diet <5 years prior to screening, any malignancy in the year prior to screening or cancer survivors >10 years, the presence of a cardiovascular disease or peptic ulcer, pregnancy, and lactation were set as exclusion criteria. Patients who changed their medication or diet or who initiated nutritional supplement intake during the trial were considered to be protocol violators. The recruitment of patients was conducted according to the above criteria in the IASO IBD Unit. A qualified and independent (unrelated to the project) gastroenterologist was responsible for all trial-related medical decisions, for ensuring that the selection of patients at baseline was unbiased, and for the unbiased follow-up assessment according to the protocol. Demographical data, medical history, including such data as the date of disease onset, duration, and medical treatment, and anthropometrics (body weight, height, body mass index) were documented.

After the baseline assessment, patients were randomly allocated to an intervention, either verum or a placebo. Randomisation was achieved by a computer-generated random number list that was prepared by an investigator with no clinical involvement in the trial. Blinding of intervention staff and participants was strictly maintained. The PL group received a natural PL supplement at a dose of 2.8 g daily (four tabs × 700 mg PL), while the placebo group received identical placebo tablets. This dose was chosen based on a previous pilot study in CD patients experiencing no side effects and a bioavailability study in healthy humans [22,26,27]. The intervention lasted 3 months. Both groups continued their usual medical treatment, which had to remain unaltered throughout the trial.

The verum tablets weighed 0.98 g and consisted of 70% PL, 14% microcrystalline cellulose, 14% dibasic calcium phosphate anhydrous, and 2% magnesium stearate. The supplement was standardised to 14 mg mastihadienonic acid and 13 mg isomastihadienonic acid. The placebo tablets weighed 0.99 g and consisted of 49% microcrystalline cellulose with a characteristic off-white to yellowish colour for similarity to verum, 49% dibasic calcium phosphate anhydrous, and 2% magnesium stearate. The verum and placebo tablets had an identical appearance and shared organoleptic characteristics.

### 2.2. Laboratory Analyses

#### 2.2.1. Evaluation of Oxidative Stress

##### The Total Serum Oxidisability Assay 

Serum samples were oxidised with copper sulfate as described by Aurrekoetxea and colleagues [35]. After dilution with phosphate-buffered saline (PBS, Arlington, TX, USA), samples were placed in the wells of a flat-bottomed plate. Copper sulfate was added at time zero and the plate was placed in an ELISA reader (Biotek PowerWave XS2, Winuski, VT, USA). The conjugated dienic hydroperoxides that were produced were measured at 245 nm. The increase in absorbance was plotted against time. The kinetics of oxidation were analysed in terms of the lag-time preceding oxidation and expressed in seconds (tLAG). All samples were measured in duplicate.

##### The oxLDL Assay

The levels of oxLDL in plasma were measured photometrically in an ELISA reader (Biotek PowerWave XS2) applying an ELISA Sandwich Kit according to the instructions of the manufacturer (Mercodia, AB, Uppsala, Sweden). Additionally, the oxLDL/HDL and oxLDL/LDL ratios were calculated. All samples were measured in duplicate.

##### The Uric Acid Assay

Uric acid (UA) was quantified in serum (Biosis, GR). The assay is based on the oxidation of UA in the presence of the enzyme uricase that results in the production of H_2_O_2_. The red-coloured product of the reaction of H_2_O_2_ with a phenolic derivative and 4-aminophenazone is proportional to the concentration of UA and was measured at 510 nm in a microplate spectrophotometer (Biotek PowerWave XS2). All samples were measured in duplicate.

#### 2.2.2. AA Analysis

Free (physiological) plasma AAs in an overnight fasting state were determined by gas chromatography-mass spectrometry (GC-MS), employing the Phenomenex^®^ EZ:faast kit (California, CA, USA). The procedure involves a solid phase extraction followed by derivatisation and subsequent liquid/liquid extraction. The derivatised AAs are then analysed by GC-MS.

An Agilent (Wallborn, Germany) series GC 6890 N gas chromatograph, coupled with an HP5973 Mass Spectrometer detector (EI, 70 eV), a split-splitess injector, and an HP7683 autosampler were used for analysis. An aliquot (2 μL) of derivatised samples was injected into the GC at a split ratio of 1:15. AA separation was achieved using a Phenomenex Zebron ZB-A AA analysis-dedicated column (length = 10 m, internal diameter = 0.25 mm, film thickness = 25 μm). The carrier gas was helium at a constant flow of 1.1 mL/min. The injector and transfer line temperatures were 250 °C and 340 °C, respectively. The initial oven temperature was 110 °C, which was then increased (30 °C/min), and held at 320 °C for 3 min. A selective ion monitoring (SIM) GC-MS method was applied for the detection of 26 AAs based on the ±0.05 retention time presence of target and qualifier ions at the predetermined ratios. The retention times and the target and qualifier ions of AAs are shown in Supplementary Table S1. Quantification was carried out employing norvaline as an internal standard and constructing reference curves for each AA by standard solutions.

### 2.3. Statistical Analysis

Continuous variables are presented with the mean and the standard deviation (SD) or with the median and the interquartile range (IQR). Quantitative variables are presented with the absolute and relative frequencies. All analyses were conducted on an intention-to-treat basis. For the comparison of proportions, chi-squared and Fisher’s exact tests were used. For the comparison of means between the control group and the intervention group, the Student’s *t*-test was computed. To reduce the bias implicit in utilising only complete cases, multiple imputation procedures for all of the data were implemented. Differences in changes of study variables during the follow-up period between the two study groups were evaluated using repeated measurements analysis of variance (ANOVA). The Kolmogorov–Smirnov test was applied to examine the normal distribution. For variables with a skewed distribution, the ranks were used for the analysis of variance. Statistical significance was set at 0.05, and all analyses were conducted using the SPSS statistical software (version 22.0).

The study had a power of 0.90 for the between-subjects main effect at an effect size of 0.37; a power of 0.95 for the within-subjects main effect at an effect size of 0.25; and a power of 0.95 for the interaction effect at an effect size of 0.25.

## 3. Results

Sixty IBD patients met the criteria for recruitment. Out of the 60 patients, 27 (45.0%) were randomised to the placebo group and 33 (55.0%) to the verum group, while 40 (66.7%) of them were diagnosed with CD and 20 (33.3%) with UC. Clinical, anthropometric, and demographic characteristics for all groups are presented in Table 1 and Table 2. No adverse events were reported in either treatment arm. Additionally, 24-h recalls were analysed applying the Nutritionist Pro software and no significant differences were observed in macronutrient or micronutrient intake between the mean changes in groups (Supplementary Material, Table S2). No significant changes were observed in the AAs of diet between the mean changes in groups either (Supplementary Material, Table S3).

Table 3 presents the oxidative stress markers before and after the intervention. The levels of oxLDL/LDL decreased significantly in CD patients, and the difference in mean changes between the placebo and intervention groups was significant. The mean changes were unaltered for lag time and uric acid; however, the lag time of serum oxidation significantly increased and this was paralleled by attenuation in the decrease of uric acid in serum. In the total sample, apart from oxLDL/LDL, the mean changes were significantly different between the control and intervention groups also for oxLDL/HDL. In UC, no significant differences were reported. The effect of therapy, as well as the effect of the type of disease (UC, CD) on changes in oxidative stress markers, was explored via repeated measurements models and no significant associations were found (*p* > 0.05).

Significant differences in levels of AAs are presented in Table 4. In CD patients, the levels of thioproline and lysine decreased in the PL arm; however, the difference in mean changes in PL and placebo remained insignificant. Nevertheless, in patients with UC, some interesting results were extracted. The levels of the following AAs were significantly decreased in UC patients receiving a placebo compared with their baseline, but remained largely unchanged in the UC patients receiving PL: allo-isoleucine, isoleucine, lysine, tyrosine, and tryptophane. Tyrosine rose significantly in the UC verum group compared with the baseline. In the cases of allo-leucine, glutamine, isoleucine, leucine, lysine, ornithine, serine, tryptophan, and tyrosine, the end-of-study levels in placebo patients were statistically distinct from the results in the verum group (*p* < 0.05 in each case). In the total sample, aspartic acid fell significantly in the PL group and glutamic acid rose significantly, but there was no significant difference between the two groups, and cysteine rose only in the verum group. No other significant changes were reported.

## 4. Discussion

Herein, the effect of a natural supplement prepared with PL on biomarkers of oxidative stress in IBD patients in relapse has been investigated. Furthermore, the AA profiles in patients as potential biomarkers of metabolic changes have been characterised. There are pre-clinical and pilot clinical data supporting PL administration in IBD [26,27,36]. The results described herein are part of a larger survey, which is to our knowledge the first one that evaluates the effectiveness of PL on the basis of a Phase-II randomised placebo-controlled clinical trial of IBD patients in relapse.

OxLDL is considered to be the most specific biomarker for oxidative stress evaluation in humans; most recently, its measurement by immunological methods (i.e., antibodies) has been recognised by the European Food Safety Authority (EFSA) as a reliable method to assess oxidative damage with appropriate specificity [37]. In this study, oxLDL fell significantly only in patients receiving PL, even if the mean change between the two groups remained insignificant. However, data on oxLDL levels in IBD patients are limited. A previous study in eight patients with active CD and eight controls showed that CD patients had elevated levels of oxLDL compared with the controls, reflecting the increased oxidative stress and the higher risk for atherosclerosis [38]. The decreased levels of oxLDL after PL intervention could point towards an antioxidant effect of this natural supplement in active IBD patients. Future research on its mechanism of action is justified, since its main bioactive compounds, which are mostly triterpenes (i.e., mastihadienonic and isomastihadeinonic acids), are bioavailable and exhibit antioxidant effects in vivo [22]. Azathioprine is an immunosuppressant drug that, despite its severe short- and long-term adverse effects, is most frequently used to induce and maintain remission in IBD. Although the number of patients treated with azathioprine was low and equal to seven for each trial arm, a subgroup analysis for patients treated with azathioprine showed no significant changes in oxidative stress markers.

Another interesting result of the present study was the significant reduction in the ratios of oxLDL/LDL and oxLDL/HDL in the PL arm compared with the placebo arm. A decrease in feacal lysozyme in the PL arm has been reported in the study of Papada and coworkers [29], and this parallels the decrease in the oxLDL/LDL and oxLDL/HDL ratios. Herein, when changes in oxidative stress were assessed separately for CD and UC patients, significant changes were reported for patients with CD, showing possibly a most promising response to this antioxidant supplement in CD compared with UC. In CD, the increased oxidative stress has been documented as a key factor of the disease’s pathogenesis [39], and evidence supports the implication of ROS in oxidative damage in peripheral blood of naïve and treated patients with CD as well as the role of antioxidant mechanisms in the regulation of cellular processes [40,41,42]. As such, elevated hydrogen peroxide levels have been correlated with inflammation in CD and oxidative damage is insistent in the immune cells of active and inactive CD patients, whereas catalase activity is permanently inhibited [40]. However, despite the indisputable presence of oxidative stress in CD and although these patients are at increased risk for cardiovascular disease [1,43], data on the above ratios are scarce. Overall, the level of oxLDL and the oxLDL/LDL and oxLDL/HDL ratios seem to be better biomarkers than total cholesterol, triglycerides, HDL-C, and LDL-C for discrimination between patients with increased cardiovascular risk and healthy subjects [43]. In addition, they seem to be the most useful clinical parameters of lipoprotein oxidation in other pathologies as well, such as type II diabetes, reflecting the association between lipids in the state of oxidative stress [44]. A study in elderly patients with type II diabetes showed that oxLDL/HDL was directly associated with advanced glycation end products and advanced oxidation protein products, which enhance oxidative stress [45]. A more recent study in a population with a high prevalence of cardiovascular disease and type II diabetes showed that these ratios were significantly associated with HbA1c, glucose, and CRP [46]. To our knowledge, this is the first study evaluating these ratios in patients with active CD and UC in the context of a randomised clinical trial that is designed to assess the effectiveness of a natural supplement. These results in CD could point towards a favourable effect of an antioxidant supplement on the mechanisms of systemic inflammation that increase the risk of cardiovascular disease.

AAs are both substrates and regulators in several metabolic pathways. Circulating AA levels may reflect not only nutritional status, but also the inflammatory state and disease activity. In this study of patients with active IBD, no significant changes in dietary AAs were reported and this was paralleled by no significantly different changes in plasma-free AAs between the groups at baseline and at follow-up. However, some stimulating results regarding AAs changes in UC have been revealed. As such, levels of allo-isoleucine, isoleucine, cysteine, and tryptophane significantly decreased in the placebo group, whereas in the PL group they remained unchanged, with the mean changes between the groups being significant. The mean changes between the two UC groups differed significantly for leucine, serine, and glutamine. Plasma-free AA normalisation paralleled the improvement in oxidative stress biomarkers; however, no significant correlation was reported (*p* > 0.05). The general tendency for plasma AAs to increase in UC patients on the placebo may indicate increased de novo AA synthesis in the presence of inflammation. The fact that AA alterations were present predominantly in the group of UC patients may indicate a more favourable effect of PL in patients with active UC.

Several AAs are implicated in oxidative stress. Cysteine is a precursor of glutathione. Cysteine was significantly lower in the placebo group and correlated negatively with levels of oxLDL (*r* = −0.26, *p* = 0.044). This is in accordance with previous findings that oxidative stress in vivo could be translated as a deficiency of glutathione and/or cysteine [47]. Tryptophane, which was significantly decreased in the placebo group, is metabolised along the oxidative kynurenine pathway leading to the generation of quinolinic acid, 3-hydroxykynurenine, and 3-hydroxyanthranilic acid, all of which are known to generate free radicals [48]. In addition, proline is upregulated as an oxidative stress response in mammalian cells [49]. Previous studies in intestinal cell culture models have shown that the presence of sulfhydryl-containing AAs, branched-chain AAs (BCAAs), heterocyclic-group-containing AAs, or other AAs is associated with attenuation of oxidative-stress-induced inflammation [50,51].

While this study has interesting results and the quality of being double-blinded, it has limitations, including the absence of endoscopy at follow-up, precluding the ability to comment on histological alterations, and the absence of an identification of the full metabolic profile. Arginine, the immediate precursor of nitric oxide, which regulates vasodilation, was not within the group of AAs targeted, limiting the knowledge on PL’s effect on vasodilation. It is also compromised by the fact that no specific diet was recommended the day before the assessment. Additionally, treatments included steroids, ASA, and immunosupressants, and patients were in relapse for different time periods. However, these limitations are compensated by the very tight control of the verum and placebo groups to ensure compliance of patients with the protocol, as well as the adequate power of the study overall, which ensued from the total number of participants that were recruited.

## 5. Conclusions

Given the increasing interest in the use of natural supplements with antioxidant and anti-inflammatory properties in IBD, herein the effects of a PL supplement on oxidative stress biomarkers in active IBD patients were explored. These results showed, for the first time, that PL could exhibit favourable effects in oxidative stress biomarkers in patients with active CD that could be of clinical importance.

## Figures and Tables

**Table 1:** Demographics in Inflammatory Bowel Disease (IBD) patients or in the Crohn’s disease (CD) and ulcerative colitis (UC) subgroups. The results are given as *N* (%) of the total number.

	IBD (*N* = 60)			CD (*N* = 40)			UC (*N* = 20)		
	Placebo (27)	PL (33)	*P*	Placebo (18)	PL (22)	*P*	Placebo (9)	PL (11)	*P*
**Sex**									
Females	9 (33.3)	18 (54.5)	0.100 ^+^	7 (38.9)	11 (50)	0.482 ^+^	2 (22.2)	7 (63.6)	0.092 ^++^
Males	18 (66.7)	15 (45.5)		11 (61.1)	11 (50)		7 (77.8)	4 (36.4)	
**Age** (years), mean (SD)	45 (17.4)	38.2 (11.9)	0.076 ^‡^	48.1 (17.6)	36 (10.7)	0.011 ^‡^	38.9 (16.1)	42.5 (13.6)	0.597 ^‡^
**Marital status**									
Unmarried/Divorced	14 (51.9)	14 (42.4)	0.466 ^+^	9 (50)	11 (50)	1.000 ^+^	5 (55.6)	3 (27.3)	0.362 ^++^
Married	13 (48.1)	19 (57.6)		9 (50)	11 (50)		4 (44.4)	8 (72.7)	
**Education (years)**									
1–9	3 (11.1)	7 (21.2)	0.510 ^+^	2 (11.1)	2 (9.1)	0.884 ^++^	1 (11.1)	5 (45.5)	0.326 ^++^
10–12	5 (18.5)	7 (21.2)		3 (16.7)	6 (27.3)		2 (22.2)	1 (9.1)	
>12	19 (70.4)	19 (57.6)		13 (72.2)	14 (63.6)		6 (66.7)	5 (45.5)	
**Smoking** ^#^									
No	20 (74.1)	23 (69.7)	0.653 ^+^	11 (61.1)	15 (68.2)	0.641 ^+^	9 (100)	8 (72.7)	0.211 ^++^
Yes	7 (25.9)	10 (30.3)		7 (38.9)	7 (31.8)		0 (0)	3 (27.3)	

^#^ Smoking habits are presented herein at baseline; ^+^ Pearson’s chi-square test; ^++^ Fisher’s exact test; ^‡^ Student’s *t*-test. PL, *Pistacia lentiscus*.

**Table 2:** Anthropometrics and clinical characteristics in IBD patients. The results are given as *N* (%) of the total number.

	IBD (*N* = 60)		*P*	CD (*N* = 40)		*P*	UC (*N* = 20)		*P*
	Placebo (27)	Mastiha (33)		Placebo (18)	Mastiha (22)		Placebo (9)	Mastiha (11)	
**BMI** (kg/m^2^) mean (SD)	24.5 (6.6)	23.4 (5.3)	0.481 ^‡^	24.4 (6.1)	22 (4.2)	0.149 ^‡^	24.7 (7.8)	26.2 (6.2)	0.628 ^‡^
Normal	17 (63.0)	23 (69.7)	0.863 ^++^	11 (61.1)	17 (77.3)	0.292 ^++^	6 (66.7)	6 (54.5)	0.830 ^++^
Overweight	5 (18.5)	6 (18.2)		3 (16.7)	4 (18.2)		2 (22.2)	2 (18.2)	
Obese	5 (18.5)	4 (12.1)		4 (22.2)	1 (4.5)		1 (11.1)	3 (27.3)	
**Disease duration** (years), mean (SD)	13.9 (10.7)	9.4 (7.0)	0.061 ^‡^	14.8 (12.0)	10.2 (6.9)	0.131 ^‡^	12.0 (7.7)	7.9 (7.4)	0.240 ^‡^
**Age of first symptoms**, mean (SD)	29.6 (16.1)	27.0 (11.9)	0.480 ^‡^	31.2 (16.6)	25.0 (10.3)	0.152 ^‡^	26.4 (15.7)	31.6 (14.2)	0.463 ^‡^
**Age of diagnosis**, mean (SD)	32.0 (17.4)	27.9 (11.9)	0.290 ^‡^	34.5 (18.0)	25.8 (10.6)	0.065 ^‡^	26.9 (15.9)	32.4 (13.9)	0.431 ^‡^
**Disease extent**									
Ileal				9 (50.0)	8 (36.4)				
Ileocolonic				7 (39.0)	9 (40.9)				
Colonic				1 (5.5)	3 (13.6)				
Colonic and duodenum				1 (5.5)	0 (0.0)				
Colonic and upper GI-				0 (0.0)	2 (9.1)				
Pancolitis							6 (66.7)	8 (72.7)	
Left-sided							3 (33.3)	2 (18.2)	
Rectitis							0 (0)	1 (9.1)	
**Surgical therapy** in the past	12 (44.4)	12 (36.4)	0.525 ^+^	10 (55.5)	10 (45.5)	0.525 ^+^	2 (22.2)	2 (18.2)	1.000 ^++^
**Concomitant treatment**									
None	9 (33.3)	12 (36.4)	0.966 ^+^	7 (38.9)	10 (45.5)	0.783 ^+^	2 (22.2)	2 (18.2)	0.850 ^++^
One	10 (37.0)	12 (36.4)		5 (27.8)	7 (31.8)		5 (55.6)	5 (45.5)	
Combined	8 (29.6)	9 (27.3)		6 (33.3)	5 (22.7)		2 (22.2)	4 (36.4)	
**Medication**									
Mesalamine	11 (40.7)	13 (39.4)	0.916 ^+^	5 (27.8)	4 (18.2)	0.705 ^++^	6 (66.7)	9 (81.8)	0.617 ^++^
Azathioprine	7 (25.9)	7 (21.2)	0.668 ^+^	5 (27.8)	4 (18.2)	0.705 ^++^	2 (22.2)	3 (27.3)	1.000 ^++^
Corticosteroids	9 (33.3)	14 (42.4)	0.807 ^+^	7 (38.9)	10 (45.5)	0.676 ^+^	2 (22.2)	4 (36.4)	1.000 ^++^

^+^ Pearson’s chi-square test; ^++^ Fisher’s exact test; ^‡^ Student’s *t*-test. BMI, body mass index.

**Table 3:** The oxidative stress markers at baseline and at follow-up. Values are expressed as the mean ± SD.

	IBD (*N* = 60)					CD (*N* = 40)					UC (*N* = 20)				
	Pre-	Post-	Change	*P* ^1^	*P* ^2^	Pre-	Post-	Change	*P* ^1^	*P* ^2^	Pre-	Post-	Change	*P* ^1^	*P* ^2^
**oxLDL (U/L)**															
Placebo	135.3 (48.38)	135.45 (38.92)	0.15 (52.76)	ns	ns	122.7 (47.1)	128.3 (43)	5.6 (50.4)	ns	ns	160.4 (42.8)	149.7 (25.4)	−10.7 (58.8)	ns	ns
PL	160.42 (34.26)	140.12 (41.91)	−20.3 (51.7)	0.031		159.4 (34.5)	138.2 (44.1)	−21.2 (55.3)	ns		162.4 (35.3)	144 (39)	−18.4 (46)	ns	
*P* ^3^	0.022	ns				0.007	ns				ns	ns			
**oxLDL/HDL**															
Placebo	2.44 (1.22)	2.63 (1.38)	0.19 (1.67)	ns	0.044	2.44 (1.2)	2.51 (1.51)	0.07 (1.63)	ns	ns	2.45 (1.33)	2.88 (1.11)	0.43 (1.83)	ns	ns
PL	3.06 (0.91)	2.37 (1.42)	−0.69 (1.63)	0.020		2.98 (0.85)	2.45 (1.42)	−0.53 (1.46)	ns		3.23 (1.03)	2.2 (1.45)	−1.03 (1.96)	ns	
*P* ^3^	0.028	ns				ns	ns				ns	ns			
**oxLDL/LDL**															
Placebo	1.62 (0.88)	1.7 (0.88)	0.07 (0.82)	ns	0.015	1.55 (0.99)	1.79 (0.96)	0.24 (0.73)	ns	0.010	1.78 (0.63)	1.51 (0.73)	−0.27 (0.92)	ns	ns
PL	1.84 (0.73)	1.3 (0.85)	−0.54 (1.03)	0.005		1.95 (0.84)	1.34 (0.94)	−0.61 (1.15)	0.006		1.62 (0.38)	1.23 (0.65)	−0.39 (0.76)	ns	
*P* ^3^	ns	ns				ns	ns				ns	ns			
**Lagtime (s)**															
Placebo	4279.8 (2573.79)	4889.15 (2094.22)	609.31 (2159.4)	ns	ns	4014.1 (2335.6)	4619.9 (1869.8)	605.8 (2210)	ns	ns	4811.3 (3075.8)	5427.6 (2516.9)	616.3 (2185.5)	ns	ns
PL	4271.12 (2236.36)	4959.46 (1760.57)	688.34 (2327.7)	ns		3823.2 (1763.5)	4852.6 (1793.3)	1029.4 (2119)	0.031		5166.9 (2854.6)	5173.2 (1757.7)	6.3 (2672.4)	ns	
*P* ^3^	ns	ns				ns	ns				ns	ns			
**UA (mg/dL)**															
Placebo	4.99 (1.92)	4.45 (1.22)	−0.54 (1.79)	ns	ns	5.25 (2.22)	4.39 (1.29)	−0.9 (2)	0.037	ns	4.46 (1.04)	4.58 (1.12)	0.1 (0.9)	ns	ns
PL	5.76 (4.9)	4.64 (1.11)	−1.12 (4.82)	ns		5.14 (1.45)	4.58 (1.15)	−0.6 (1.4)	ns		7.01 (8.36)	4.76 (1.08)	−2.3 (8.3)	ns	
*P* ^3^	ns	ns				ns	ns				ns	ns			

^1^*p*-value for the time effect; ^2^ The effects reported include differences between the groups in the degree of change (repeated measurements ANOVA); ^3^ *p*-value for the group effect; ns, non-significant.

**Table 4:** Significant differences in amino acids (AAs) levels (nmoL/mL). Values are expressed as the mean (SD) or median (interquartile range, IQR).

	Pre-	Post-	Change	*P* ^1^	*P* ^2^
**IBD (*N* = 60)**					
**Aspartic acid**					
Placebo	7 (4.1)	6.7 (4.2)	−0.3 (6.2)	ns	ns
PL	8.2 (4.5)	5.3 (3.3)	−2.9 (6)	0.009	
*P* ^3^	ns	ns			
**Cysteine ^‡^**					
Placebo	27.9 (26.5; 28.8)	27.5 (26.3; 29.6)	0 (−2; 2.1)	ns	ns
PL	27.1 (25.4; 28.5)	28.7 (26.2; 29.5)	1.3 (−0.7; 3.5)	0.042	
*P* ^3^	ns	ns			
**Glutamic acid ^‡^**					
Placebo	12.3 (5.1; 26.5)	20.2 (9.5; 30)	3.6 (−7.4; 15.1)	0.039	ns
PL	18.6 (10.4; 26.6)	25.7 (21.7; 28.2)	4 (−3.6; 16.3)	0.045	
*P* ^3^	ns	0.042			
**Glutamine ^‡^**					
Placebo	433.9 (392.9; 461.8)	385 (375.1; 446.4)	−34.4 (−60.4; −13.8)	ns	ns
PL	368.4 (303.1; 428.4)	385.4 (311; 413.1)	−7.4 (−53.7; −67.6)	ns	
*P* ^3^	0.011	ns			
**Lysine ^‡^**					
Placebo	185.3 (160.1; 222.3)	171.3 (153.4; 177.1)	−25.8 (−58.5; −0.9)	ns	ns
PL	188.1 (163.3; 220.8)	164.3 (150.3; 172.2)	−27 (−55.8; 1.9)	ns	
*P* ^3^	ns	ns			
**Thioproline**					
Placebo	16.7 (4.1)	14.1 (2.3)	−2.7 (5.2)	0.006	ns
PL	18.1 (4.2)	13.7 (2.8)	−4.4 (4.6)	<0.001	
*P* ^3^	ns	ns			
**CD (*N* = 40)**					
**Cysteine ^‡^**					
Placebo	28.3 (26.9; 29.3)	27.2 (26.3; 29.3)	−0.8 (−2.2; 1.7)	ns	ns
PL	27.2 (25.6; 28.5)	27.7 (25; 29.2)	0.2 (−1.9; 3.7)	ns	
*P* ^3^	0.050	ns			
**Lysine ^‡^**					
Placebo	165.3 (142.8; 218.4)	173.4 (155; 178.2)	−9.9 (−50.1; 1.5)	ns	ns
PL	192.6 (175.3; 221.6)	160.8 (142.7; 171.4)	−30.6 (−62.2; −4)	0.036	
*P* ^3^	ns	ns			
**Thioproline**					
Placebo	15.6 (3.6)	14.4 (2.4)	−1.2 (4.9)	ns	ns
PL	17.8 (4.8)	13.4 (3.1)	−4.4 (5)	<0.001	
*P* ^3^	ns	ns			
**UC (*N* = 20)**					
**Alo-isoleucine ^‡^**					
Placebo	58.4 (53; 68.9)	48.7 (41.4; 55.5)	−14.4 (−15.4; −11.6)	0.038	0.016
PL	45.5 (32; 52.4)	51.8 (47.3; 55.6)	7.9 (−4.2; 17.2)	ns	
*P* ^3^	0.007	ns			
**β-Aminobutyric acid ^‡^**					
Placebo	98.1 (96.8; 99.9)	98 (97; 98.8)	−1.1 (−2.5; −0.4)	ns	ns
PL	98.7 (97.3; 99.4)	98.8 (98.1; 101.7)	1 (−0.9; 3.8)	ns	
*P* ^3^	ns	0.022			
**Aspartic acid**					
Placebo	6.4 (2.4)	5.6 (2.5)	−0.8 (4.4)	0.672	ns
PL	9.7 (4.6)	5.6 (3.5)	−4.1 (5.6)	0.016	
*P* ^3^	ns	ns			
**Cysteine ^‡^**					
Placebo	27.7 (26.5; 27.9)	28.8 (26.5; 29.6)	1.2 (0.2; 2.3)	ns	ns
PL	26.4 (25.1; 28.6)	29.2 (28.1; 31.5)	2.1 (0.8; 3.5)	<0.001	
*P* ^3^	ns	ns			
**Glutamic acid ^‡^**					
Placebo	4.1 (2.9; 17.1)	14.9 (9.1; 19.6)	2.2 (−2.8; 6.6)	ns	ns
PL	21.1 (10.4; 34.1)	24.4 (21.7; 30.3)	2.2 (−3; 19.9)	ns	
*P* ^3^	ns	0.005			
**Glutamine ^‡^**					
Placebo	427.2 (401.8; 456.3)	385 (375.1; 394.7)	−37.5 (−47.9; −22.7)	ns	0.038
PL	330.6 (255.4; 428.4)	387.1 (311; 413.1)	50.3 (−50.2; 129.8)	ns	
*P* ^3^	0.048	ns			
**Isoleucine ^‡^**					
Placebo	68.3 (61.8; 80.2)	51.5 (49.1; 63.6)	−16.9 (−17.6; −10.4)	0.019	0.009
PL	55.4 (38.1; 61.5)	60.4 (55.2; 67.8)	0.8 (−3.3; −24.4)	ns	
*P* ^3^	0.008	ns			
**Leucine ^‡^**					
Placebo	132.3 (115.2; 146.6)	117.7 (92.3; 139.3)	−23.8 (−29.9; −7.9)	ns	0.043
PL	102.1 (85; 123.7)	127.8 (120.1; 137.3)	16.1 (0.7; 42.8)	ns	
*P* ^3^	ns	ns			
**Lysine ^‡^**					
Placebo	207.2 (190.5; 222.3)	164.1 (153.4; 174.3)	−47.5 (−69.1; −41.6)	0.002	0.009
PL	163.3 (129.9; 209.9)	166.7 (159.5; 182.8)	−0.6 (−33.4; 26.5)	ns	
*P* ^3^	ns	ns			
**Ornithine ^‡^**					
Placebo	103.5 (77.8; 125.5)	75.2 (69.1; 82.9)	−20.8 (−50.3; −3.8)	ns	0.036
PL	77.1 (47.1; 96.1)	85 (68.5; 92.9)	15.2 (−27.6; 22.4)	ns	
*P* ^3^	ns	ns			
**Proline**					
Placebo	257.9 (55.7)	223.6 (56.1)	−34.3 (92.2)	ns	ns
PL	240.6 (90.9)	275.7 (49.6)	35.1 (114.8)	ns	
*P* ^3^	ns	0.041			
**Serine**					
Placebo	130.4 (36.2)	114.1 (19.8)	−16.3 (32.8)	ns	0.028
PL	94.8 (28.1)	108.3 (15.3)	13.5 (23)	ns	
*P* ^3^	0.023	ns			
**Thioproline**					
Placebo	19.1 (4.4)	13.3 (1.8)	−5.8 (4.8)	0.001	0.047
PL	18.7 (2.7)	14.1 (2.1)	−4.6 (4)	0.003	
*P* ^3^	ns	ns			
**Tryptophane**					
Placebo	65.3 (12.3)	57 (12.8)	−8.3 (12)	0.050	0.037
PL	55.1 (13.9)	58.8 (9)	3.7 (11.7)	ns	
*P* ^3^	ns	ns			
**Tyrosine**					
Placebo	71 (18.4)	58.1 (17.2)	−12.9 (17.3)	0.045	0.005
PL	50.4 (18.6)	63.3 (11.9)	12.9 (18.6)	0.029	
*P* ^3^	0.023	ns			

^1^*p*-value for the time effect; ^2^ The effects reported include differences between the groups in mean changes (repeated measurements ANOVA); ^3^ *p*-value for the group effect; ^‡^ The analyses were based on ranks, and variables are presented with the median (IQR); ns, non-significant.

## Supplementary Materials

The following are available online at http://www.mdpi.com/2072-6643/10/11/1779/s1, Supplementary Table S1: Retention times and target and qualifier ions of the AAs and the internal standard. Supplementary Table S2: Nutritional intake in IBD patients in relapse before and after intervention. Values are median (IQR). Table S3: Dietary AAs (mg) in IBD patients in relapse before and after intervention. Values are median (IQR).
