# Supplementary material for: Antioxidative Efficacy of a Pistacia Lentiscus Supplement and Its Effect on the Plasma Amino Acid Profile in Inflammatory Bowel Disease: A Randomised, Double-Blind, Placebo-Controlled Trial
Source: Nutrients. 2018 Nov 16;10(11):1779. doi: 10.3390/nu10111779 (PMC6267573; doi:10.3390/nu10111779)
Supplement: Supplementary file 1 [file nutrients-10-01779-s001.pdf]

nutrients

MDPI

Article

Antioxidative Efficacy of a *Pistacia Lentiscus* Supplement and its Effect on the Plasma Amino Acid Profile in Inflammatory Bowel Disease: A Randomised, Double-Blind, Placebo-Controlled Trial

Efstathia Papada [1], Alastair Forbes [2], Charalampia Amerikanou [1], Ljilja Torović [3], Nick Kalogeropoulos [1], Chara Tzavara [1], John K. Triantafillidis [4], and Andriana C. Kaliora [1,*]

Supplementary Materials

Supplementary Table S1. Retention times, target and qualifier ions of the amino acids and internal standard.

Amino acid	Rt (min)	Target Ion (m/z)	Qualifier Ions (m/z)
Alanine	1.161	130	88
Glycine	1.261	116	102
α-Aminobutyric acid	1.366	144	102
Valine	1.462	158	116
β-Aminoisobutyric acid	1.534	116	143, 172
Norvaline (Internal Standard)	1.590	158	72
Leucine	1.675	172	86
Allo-isoleucine	1.703	172	130
Isoleucine	1.731	172	130
Threonine	1.943	160	101
Serine	1.980	156	203
Proline	2.051	156	243
Asparagine	2.150	155	69
Thioproline	2.719	174	147
Aspartic acid	2.723	216	130
Methionine	2.742	203	277
Hydroxyproline	2.879	172	130
Glutamic acid	3.081	230	170
Phenylalanine	3.100	206	190
α-Aminoadipic acid	3.396	244	98
Glutamine	3.720	187	84
Ornithine	4.134	156	70
Lysine	4.407	170	128
Histidine	4.585	282	168
Tyrosine	4.877	206	107
Tryptophan	5.959	130	
Cysteine	5.897	248	216

Supplementary Table S2. Nutritional intake in IBD patients in relapse before and after intervention. Values are median (IQR).

	**Baseline**	**Follow-up**	**Change**		
	Median (IQR)	Median (IQR)	Median (IQR)	*P* [1]	*P* [2]
**Energy consumption (kcal/day)**					
Placebo	1768.6 (1133.5 ; 2151.4)	1676.1 (1134 ; 1859.7)	-57.2 (-634.5 ; 496.6)	ns	ns
PL	1815 (1531.5 ; 2231.5)	1811.6 (1554.2 ; 2155)	-59 (-497.7 ; 198.4)	ns	
P [3]	ns	ns			
**Protein (g/day)**					
Placebo	70.6 (49.6 ; 89.6)	75.8 (20.6 ; 95.3)	-8.1 (-55.5 ; 31.1)	**0.041**	ns
PL	80.7 (61.6 ; 118.7)	92.3 (60.7 ; 123.1)	7.8 (-29.7 ; 28.2)	ns	
P [3]	ns	0.045			
**Carbohydrates (g/day)**					
Placebo	198.6 (148.6 ; 259.8)	173 (140.7 ; 222.6)	-35.2 (-108.6 ; 26)	ns	ns
PL	192.2 (113.4 ; 239.9)	158.8 (77.9 ; 179.6)	-41.1 (-103.6 ;58.7)	**0.032**	
P [3]	ns	ns			
**Dietary fiber (g/day)**					
Placebo	11.4 (5.1 ;14.8)	12.7 (7.2 ;16.8)	2.9 (-4.3 ; 7.4)	ns	ns
PL	11.5 (8.7 ; 18.3)	11 (9 ; 14.6)	-0.9 (-5.9 ; 4.8)	ns	
P [3]	ns	ns			
**Sugars (g/day)**					
Glucose (g/day)					
Placebo	6.4 (2.5 ; 10.1)	8.1 (5.2 ; 14)	0.6 (-1.2 ; 6.5)	ns	ns
PL	8 (2.9 ; 11.6)	9.5 (5.3 ; 12.5)	0.1 (-3.1 ; 6.5)	ns	
P [3]	ns	ns			
Lactose (g/day)					
Placebo	0.7 (0.01 ; 4)	0.02 (0 ; 1.6)	-0.41 (-2.27 ; 0.02)	ns	ns
PL	0.02 (0 ; 5.85)	1.35 (0.01 ; 4.21)	-0.01 (-1.98 ; 2.35)	ns	
P [3]	ns	**0.022**			
Fructose (g/day)					
Placebo	8.7 (2.5 ; 15.9)	10.4 (7.6 ; 15.4)	1.9 (-4.8 ;9.8)	ns	ns
PL	8 (3.6 ; 12.3)	12 (6.4 ; 14.8)	3.2 (-2.2 ; 8.7)	ns	
P [3]	ns	ns			
**Total fat (g/day)**					
Placebo	75 (39.2 ; 98)	51.1 (32.5 ; 71.9)	-21.4 (-53.6 ; 2.2)	**0.018**	ns

	**Baseline**	**Follow-up**	**Change**		
	Median (IQR)	Median (IQR)	Median (IQR)	*P* [1]	*P* [2]
PL	67.6 (47.3 ; 86.8)	66.3 (52.7 ;76.6)	-12.1 (-29.5 ;16)	ns	
P [3]	ns	ns			
**Saturated Fatty Acids (g/day)**					
Placebo	21.8 (12.6 ; 32.7)	22 (13.3 ; 28.6)	-5.5 (-15.6 ; 7.2)	ns	ns
PL	18.3 (14.3 ; 36.2)	25.8 (19.8 ; 29.4)	0.5 (-8 ; 6.3)	ns	
P [3]	ns	ns			
**Monosaturated Fatty Acids (g/day)**					
Placebo	28.7 (18.7 ; 44.2)	23.6 (14.9 ; 31.9)	-4 (-19 ; 4.9)	ns	ns
PL	28.5 (19.5 ; 41.3)	26.1 (16.3 ; 30.5)	-9.7 (-15.7 ; 5.4)	ns	
P [3]	ns	ns			
**Polyunsaturated Fatty Acids (g/day)**					
Placebo	8.7 (6.7 ; 14.6)	8.7 (5.6 ; 10.2)	-1.8 (-5.7 ; 0.5)	ns	ns
PL	9.3 (7.5 ; 13.3)	9.6 (6.6 ; 13.8)	-1 (-3.6 ; 3.7)	ns	
P [3]	ns	ns			
**Trans Fatty Acids (g/day)**					
Placebo	0.11 (0.01 ; 0.44)	0.06 (0.01 ; 0.52)	0 (-0.39 ; 0.41)	ns	ns
PL	0.09 (0.03 ; 0.93)	0.14 (0.03 ; 0.55)	-0.04 (-0.18 ; 0.16)	ns	
P [3]	ns	ns			
**α-carotene (µg/day)**					
Placebo	5.8 (0 ; 114)	42.9 (1.7 ; 520.2)	29 (-0.8 ; 107.3)	ns	ns
PL	63.7 (2.9 ; 140)	144 (1.4 ; 568.3)	95.6 (-29.5 ; 519.2)	ns	
P [3]	ns	ns			
**β-carotene (µg/day)**					
Placebo	436.8 (61.6 ; 1342.2)	327.9 (96.6 ; 1995)	151 (-263.3 ; 1633.6)	ns	ns
PL	427.9 (124.1 ; 1131.7)	912.2 (159.7 ; 2095.9)	243.9 (-45.9 ; 1868.3)	ns	
P [3]	ns	ns			
**Vitamin D (µg/day)**					
Placebo	1.37 (0.36 ; 3.25)	0.98 (0.42 ; 2.16)	-0.24 (-1.83 ; 0.96)	ns	ns
PL	1.44 (0.68 ; 3.36)	2.68 (0.87 ; 3.56)	0.96 (-1.09 ; 2.92)	ns	
P [3]	ns	**0.002**			
**Vitamin E (mg/day)**					
Placebo	1.15 (0.22 ; 2.01)	0.72 (0.07 ; 2.73)	0 (-1.1 ; 2.1)	ns	ns

	**Baseline**	**Follow-up**	**Change**		
	Median (IQR)	Median (IQR)	Median (IQR)	*P* [1]	*P* [2]
PL	0.54 (0.33 ; 1.39)	0.98 (0.18 ; 3.21)	0.54 (-0.4 ; 2.09)	ns	
P [3]	ns	ns			
**Vitamin K (μg/day)**					
Placebo	38.8 (11.9 ; 58)	37.9 (16.8 ; 67.3)	1 (-23.3 ; 14.4)	ns	ns
PL	29.4 (21.4 ; 50)	41.6 (15 ; 98.6)	8 (-18.5 ; 43.7)	ns	
P [3]	ns	ns			
**Vitamin C (mg/day)**					
Placebo	23.5 (14.2 ; 46.6)	51.5 (21 ; 100.6)	9.5 (-11.2 ; 82.1)	ns	ns
PL	32.1 (9.5 ; 69.8)	56.5 (27.4 ; 112.5)	11.5 (-16.9 ; 43)	ns	
P [3]	ns	ns			

Ranks of the variables were used in all of the analyses. [1] *p*-value for time effect; [2] Effects reported include differences between the groups in the degree of change (repeated measurements ANOVA); [3] *p*-value for group effect.

Supplementary Table S3. Dietary AAs (mg) in IBD patients in relapse before and after intervention. Values are median (IQR).

	**Baseline Median (IQR)**	**Follow-up Median (IQR)**	***P*** [1]	***P*** [2]
**Alanine**				
Placebo	1594.8 (1272.8 ; 1942.7)	1589 (968.2 ; 2766.4)	ns	ns
PL	2088.7 (296.2 ; 3031.6)	2026.9 (702.5 ; 4813.8)	ns	
P [3]	ns	ns		
**Arginine**				
Placebo	1918.2 (1404.4 ; 2256.4)	1812.7 (1116.5 ; 3139)	ns	ns
PL	2328.6 (306.8 ; 3464.5)	2359.9 (905.7 ; 5032.6)	ns	
P [3]	ns	ns		
**Aspartic Acid**				
Placebo	2957.8 (2408.1 ; 3939.1)	3263.3 (1727.4 ; 5266.6)	ns	ns
PL	3522.6 (527.1 ; 5856.4)	3389 (2148.3 ; 8956)	ns	
P [3]	ns	ns		
**Cysteine**				
Placebo	559.6 (449.9 ; 800.4)	581.3 (462.7 ; 836.4)	ns	ns
PL	649.8 (173.1 ; 882.6)	905.3 (559.8 ; 1396)	ns	
P [3]	ns	ns		
**Glutamic Acid**				
Placebo	8001.5(6114;11114.1)	9314.3 (6277.7 ; 11856.4)	ns	ns
PL	8739.2(3000.1;13919.9)	7771 (5564.3 ; 18294.2)	ns	
P [3]	ns	ns		
**Glycine**				
Placebo	1345.8 (979.2 ; 1572)	1226.9 (837.8 ; 2323.8)	ns	ns
PL	1930.9 (295.4 ; 2696.2)	1635 (705.2 ; 3837)	ns	
P [3]	ns	ns		
**Histidine**				
Placebo	1134 (876.1 ; 1514.8)	1328.9 (687.4;1759.6)	ns	ns
PL	1342.5 (182.2 ; 2025.1)	1456.5 (558.4 ; 2800.4)	ns	
P [3]	ns	ns		
**Isoleucine**				
Placebo	1674 (1476.9 ; 2204.2)	1872.6 (1281 ; 2572.2)	ns	ns
PL	2074.4 (320.1 ; 3151.2)	1941.2 (938.7 ; 4625.9)	ns	
P [3]	ns	ns		
**Leucine**				
Placebo	2803.8 (2315.3 ; 4207.9)	3707.1 (2292.8 ; 4447.7)	ns	ns
PL	3205.4 (563.8 ; 5668.2)	3428 (1737.1 ; 7883)	ns	
P [3]	ns	ns		
**Lysine**				
Placebo	2470.9 (1677.7 ; 3830.6)	2715.8 (1567.7 ; 4093.9)	ns	ns
PL	2906.4 (236.2 ; 5090.6)	3642.1 (1251.8 ;7229)	ns	
P [3]	ns	ns		
**Methionine**				

Placebo	841.3 (681.1 ; 1244.5)	920.8(516;1256.2)	ns	ns
PL	1026.6 (142.4 ; 1615.8)	1026(471.9;2386.4)	ns	
P [3]	ns	ns		
**Phenylalanine**				
Placebo	1640.4 (1374 ; 2413.3)	2127.5 (1417.3 ; 2704.1)	ns	ns
PL	1871.5 (406.2 ; 3127.7)	1910.6 (1047 ; 4274.8)	ns	
P [3]	ns	ns		
**Proline**				
Placebo	3040.4 (1795.5 ; 4324.1)	3656.6 (2145.4 ; 4341.6)	ns	ns
PL	2576.6 (841.5 ; 5547.8)	2414.2 (2128.5 ; 5740.4)	ns	
P [3]	ns	ns		
**Serine**				
Placebo	1736.9 (1252.1 ; 2637.2)	2247.9 (1409.8 ; 2757.3)	ns	ns
PL	1721.5 (399 ; 3478.1)	1906.5 (1034 ; 4332.3)	ns	
P [3]	ns	ns		
**Threonine**				
Placebo	1433.1 (1153.6 ; 1783.9)	1583.8 (1055.7 ; 2311.4)	ns	ns
PL	1663.2 (258.2 ; 2890.1)	1692.1 (819.1 ; 3965.3)	ns	
P [3]	ns	ns		
**Tryptophan**				
Placebo	477.5 (390.9 ; 604.1)	543.1 (347.7 ; 710.2)	ns	ns
PL	504.3 (96.4 ; 821.7)	516.3 (285.9;1210.7)	ns	
P [3]	ns	ns		
**Tyrosine**				
Placebo	1327.6 (1082.9 ; 2203.2)	1568.4 (874.5 ; 1901.1)	ns	ns
PL	1362.1 (237.2 ; 2673)	1574.6 (789 ; 3491.9)	ns	
P [3]	ns	ns		
**Valine**				
Placebo	1900.4 (1492.3 ; 2832.6)	2634.2 (1477 ; 2873)	ns	ns
PL	2092 (357.1 ; 3793.4)	2186.5 (1336.8 ; 5367.1)	ns	
P [3]	ns	ns		

Ranks of the variables were used in all of the analyses; [1] *p*-value for time effect; [2] Effects reported include differences between the groups in the degree of change (repeated measurements ANOVA); [3] *p*-value for group effect.
